# Multistakeholder Participation in Disaster Management—The Case of the COVID-19 Pandemic

**DOI:** 10.3390/healthcare9020203

**Published:** 2021-02-13

**Authors:** Sigamani Panneer, Komali Kantamaneni, Robert Ramesh Babu Pushparaj, Sulochana Shekhar, Lekha Bhat, Louis Rice

**Affiliations:** 1 Department of Social Work, School of Social Sciences and Humanities and Centre for Happiness, Central University of Tamil Nadu, Thiruvarur, Tamilnadu 610005, India; sigamanip@cutn.ac.in; 2 Faculty of Creative Industries, Architecture and Engineering, Solent University, Southampton SO14 0YN, UK; 3 Department of Civil, Environmental & Geomatic Engineering, Chadwick Building, University College London (UCL), Gower St, London WC1E 6BT, UK; 4 Research Scholar, Department of Social Work, Central University of Tamil Nadu, Thiruvarur, Tamilnadu 610005, India; robertrb19@students.cutn.ac.in; 5 Department of Geography, School of Earth Sciences, Central University of Tamil Nadu, Thiruvarur, Tamilnadu 610005, India; sulochana@cutn.ac.in; 6 Department of Epidemiology & Public Health, School of Life Sciences, Central University of Tamil Nadu, Thiruvarur, Tamilnadu 610005, India; lekhabhatd@gmail.com; 7 Centre for Architecture and Built Environment Research, University of the West of England, Bristol BS16 1QY, UK; Louis.Rice@uwe.ac.uk

**Keywords:** COVID-19, multistakeholder participation, networking/collaboration, spatial decision support system, disaster management

## Abstract

The coronavirus disease 2019 (COVID-19) pandemic is affecting society’s health, economy, environment and development. COVID-19 has claimed many lives across the globe and severely impacted the livelihood of a considerable section of the world’s population. We are still in the process of finding optimal and effective solutions to control the pandemic and minimise its negative impacts. In the process of developing effective strategies to combat COVID-19, different countries have adapted diverse policies, strategies and activities and yet there are no universal or comprehensive solutions to the problem. In this context, this paper brings out a conceptual model of multistakeholder participation governance as an effective model to fight against COVID-19. Accordingly, the current study conducted a scientific review by examining multi-stakeholder disaster response strategies, particularly in relation to COVID-19. The study then presents a conceptual framework for multistakeholder participation governance as one of the effective models to fight against COVID-19. Subsequently, the article offers strategies for rebuilding the economy and healthcare system through multi-stakeholder participation, and gives policy directions/decisions based on evidence to save lives and protect livelihoods. The current study also provides evidence about multidimensional approaches and multi-diplomatic mechanisms during the COVID-19 crisis, in order to examine dimensions of multi-stakeholder participation in disaster management and to document innovative, collaborative strategic directions across the globe. The current research findings highlight the need for global collaboration by working together to put an end to this pandemic situation through the application of a Multi-Stakeholder Spatial Decision Support System (MS-SDSS).

## 1. Introduction

The world is facing the coronavirus disease 2019 (COVID-19) pandemic, which is having an unprecedent effect on people’s lives and livelihoods, leading to severe and long-term impacts at individual, community and societal levels. The pandemic crisis involves not only health issues but also economic issues [1]. Pandemics are not new to human society; however, their nature, intensity and the way societies respond change over time. In history, we have seen the most devastating pandemic, called the “black death”, which shook the world from the years 1347 to 1352 and took the lives of more than 75,000,000 people [2]. In the years 1918 to 1920, there was another pandemic called the “Spanish Flu”, where more than 100,000,000 people died [3]. Pandemics create uncertainty, complexity in understanding and there is need for new knowledge. In order to access new knowledge, it is important that we integrate the best available knowledge and reconcile often conflicting values and viewpoints. There is a need to find solutions to dealing with complicated, wicked problems such as COVID-19 that will involve complex interactions between technological, social, environmental, behavioural, managerial and medical worlds; one such strategy is multi-stakeholder participation [4], and we propose this can be combined with Multi-stakeholder Spatial Decision Support systems (MS-SDSS). The aim is to help the world to be prepared for future problems and challenges that include pandemics [5].

As the impact of the COVID-19 pandemic is multidimensional, affecting all spheres of life and across the global population, no single agency or stakeholder can work alone to control COVID-19 effectively and mitigate its impact. In order to better respond to and manage the COVID-19 situation, we need to deploy appropriate multi-stakeholder management strategies which can improve the effectiveness and efficiency of crisis and humanitarian operations [6]. It is important that competencies are developed at all levels for emergency, crisis prevention and management. COVID-19 is partly a spatial problem, highlighting the importance of quarantine, segregation and isolation in homes, workplaces and cities [7,8]. Controlling and managing these spatial issues requires an integrated, scientific approach that can help in the aggregation of spatial and non-spatial data, quick visualisation of epidemic information, spatial tracking of confirmed cases, estimation of regional transmission, and provide solid spatial information support for decision-making, measures formulation, and effective assessment of COVID-19 prevention and control measures [9,10]. 

In order to fight against COVID-19, the paper presents a conceptual model for multi-stakeholder governance which includes various stakeholders, their strategies and, in particular, a multi-stakeholder spatial decision support system. At present, there are no bespoke models of multi-stakeholder participation for dealing with COVID-19 that combine the advantages of a multi-stakeholder spatial decision support system. The model is developed based on directly and indirectly relevant research articles on disaster management; this is relevant because COVID-19 is a disaster and requires focused on multi-stakeholder participation. The paper aims to understand the implication of COVID-19 on health and development and specifically explore the most effective role for multi-stakeholder participation, as part of managing the epidemic response. However, it is acknowledged that the research only provides a snap shot of the possibilities and challenges related to multi-stakeholder participation, as the pandemic is still ongoing globally, and various stakeholders are altering, upgrading and/or updating their strategies. The paper presents a conceptual model which would be a great help towards controlling the COVID-19 pandemic and deal with its long-term impacts most effectively, and which contributes both theoretically and managerially to knowledge in this area. This helps agencies, governments and other stakeholders involved in fighting against COVID-19, with an aim to inspire researchers to take up empirical enquiries and policy makers to define polices and strategies to fight the pandemic. 

## 2. Synthesis of Literature Review 

### 2.1. Multi-Stakeholder Participation

The role of multi-stakeholders participation in this context is to work towards policy decision-making and action on global development issues, distribution of commitment and responsibility among themselves, to bring about collective action solutions for public benefit, establishing clear roles and responsibilities, getting involved in proactive prevention activities and working for community governance [11]. One of the operational definitions of multi-stakeholder participation is that it involves participation among vested-interest groups as a core activity. Multi-stakeholder collaboration may improve service delivery and participation at international, national, local/regional and community levels [12]. However, multi-stakeholder participation in decision analysis tends to become quite complex, and the outcomes can be both positive and negative. To minimise the negative results and maximise the positive outcomes, a systematic, holistic approach is required, where every stakeholder has a role to play and contribute significantly to the decision-making process [13]. 

### 2.2. Interlinking of Multi-Stakeholder Spatial Decision Support System (MS-SDSS)

**Figure 1:**
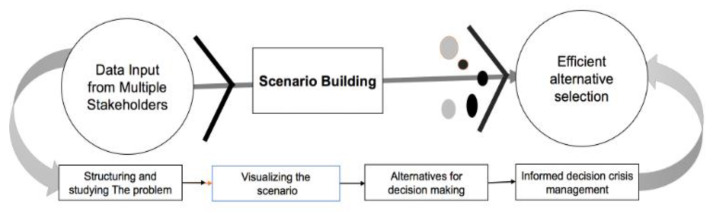
Conceptual design of Multi-Stakeholder Spatial Decision Support System (MS-SDSS)**.**

To address COVID-19, it is important to face the challenges from an interdisciplinary approach, with proactive planning, international solidarity and a global perspective [7,14,15]. For Multi-stakeholder participation, an important issue is exploring the possibility of adoption of MS-SDSS to address the challenges caused by the pandemic. The spatial decision support system (SDSS) is a computer-based information system designed to support policymakers and practitioners in decision-making processes [16]. In this context, SDSS offers a platform for the interaction of public health officials, affected actors, and first responders to improve estimations of disease propagation and likelihoods of new outbreaks [7]. Understanding the spatiotemporal dynamics of COVID-19 is essential for its mitigation, as it helps to clarify the extent and impact of the pandemic and can aid decision making, planning and community action. SDSS helps to integrate the science, data and models with decision-making at different levels of operations, policy and governance in a sustainable way over the long term [17]. A multi-stakeholder decision support system, containing data, models, tools, and a design process can assist local authorities in preparing an integrated plan for fighting COVID-19 (Figure 1); in order to facilitate multi-stakeholder planning, a design process (Figure 1) is required [18].

In this design, the multiple stakeholders are involved in the decision-making process from the problem structuring stage to the scanning of alternatives to identify the efficient one stage, followed by the last stage a set of well-studied and carefully selected efficient alternatives [19]. 

## 3. Methodology

### Review of Global Methodological Procedures

**Figure 2:**
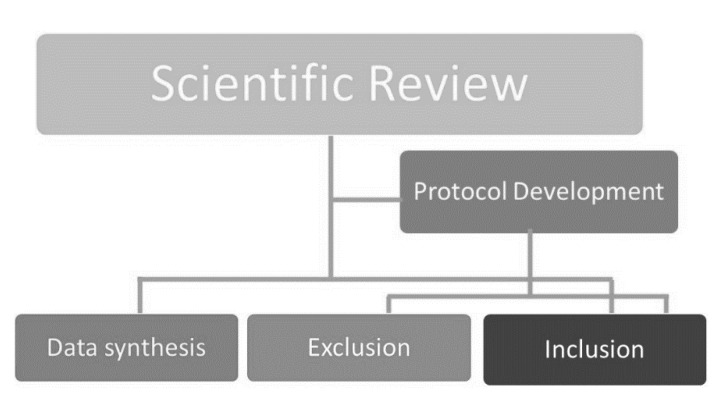
Schematic representation of methodological procedures.

There are so many methodologies (explained in Section 1 and Section 2) to review the literature globally, particularly in the multi-stakeholder participation in disaster management fields. However, we selected the scientific review methodological procedures for the current study. Accordingly, a fundamental concept of methodology has been taken from Kantamaneni [20] and subsequently applied to the current study. The present scientific literature review critically explores multi-stakeholder disaster response strategies more broadly and COVID-19 MS responses more specifically. In order to carry out the scientific literature review, first, a protocol was developed for the inclusion and exclusion criteria, then literature review and meta-analyses were identified and subsequently analysed (Figure 2).

The current study searches the various search engines and data sources: ADB (Asian Development Bank), BioSci (BioScience), BMC (Biomed Central), Elsevier journals, Future, HSB (Harvard School of Business), JAMA (Journal of the American Medical Association), JPHP (Journal of Public Health Policy), Lancet, MDPI (Multidisciplinary Digital Publishing Institute) journals, Nature, NIH (National Institutes of Health) public access, Policy Sc. (Policy Science), PubMed (Public/Publisher MEDLINE) papers, Science Adv (Science Advances), Springer journals, Google scholar, Sustain Sci (Sustainability Science ), UNDP (United Nations Development Programme) report, UN (United Nations) reports, WHO (World Health Organization) reports and Wiley publications (open access). The keywords that were used to identify the relevant data/case are: COVID-19, multistakeholder participation, disaster management, crisis management, networking, collaborations, global health, governance, crisis management, multidimensional healthcare, civil society organizations and bilateral and multi-lateral organizations. After the search, 825 various literature sources were identified; however, a review then found that some of those were not relevant to the current study. Accordingly, 432 sources were excluded from the analysis after initial screening. Then, the remining 403 articles were scrutinised for the second screening. After detailed and careful consideration, 220 more papers were excluded from the analysis. Finally, 183 were considered for full analysis. 

The inclusion criteria comprised of selecting, empirically peer-reviewed studies, peer-reviewed reviews and conceptual frameworks which were related to disaster management and COVID-19. The exclusion criteria included non-peer-reviewed papers, studies on multi-stakeholder participation that were not related to disaster management, and COVID-19 studies which were not published in the English language and studies not exactly related to the current study. While analysing the literature, we found that there were a limited number of empirical studies on multi-stakeholder participation in disaster management that involved pandemic crisis and limited availability of data. There is further lack of data as not all countries have data on multi-stakeholder participation and a lack of impact assessment studies on multi-stakeholder participation. The research presents the approaches, methods and strategies followed by different countries as case syntheses in handling COVID-19 based on the available literature at the time of writing this paper. There is a strong possibility that some countries might change their methodology and strategies as the COVID-19 situation is still on-going. Most importantly the research lacks empirical evidence on multi-stakeholder participation during COVID-19.

## 4. Scientific Review Results

### 4.1. Taxonomy of COVID-19

The COVID-19 pandemic has spread world-wide very rapidly and aggressively within a very short timeframe. Countries across the globe have adopted various prevention and control measures to minimise negative health impacts [21]. The COVID-19 pandemic and the associated economic and social crisis are posing huge challenges, including, but not limited to, availability of accurate information, free/affordable COVID-19 testing and treatment emerging issues related to jobs and income of millions of people, lack of social safety-net programs, lack of income support schemes, increased burden on women to manage family problems and the plight of migrants and informal sector workers, which are some of the important effects of this pandemic on human lives [22]. Other serious challenges relate to the restriction on economic activities, commercial, services and industrial production, the inability of firms to sell their goods and services, leading to high economic and social costs around the world due to social distancing, lockdown and quarantine [23]. More than 30 million people could fall into poverty in the absence of active policies to protect or substitute income flow to vulnerable populations [24]. 

The major societal impacts of COVID-19 include health inequality [25,26], social stratification [27], low-income individuals disproportionally affected [28] and lack of access to essential healthcare services [29]. There are gender issues like the dual burden on work, limitations of working from home and an increase in domestic violence and violation of human rights. There are increases in individual isolation, which may result in a reduction of human happiness and mental wellbeing, potentially leading to rises in psychological issues including suicide, grief, survival, and fear [24]. There are major effects on the economy including the informal sector [30], fear of losing one’s job [31], pay cuts [23], pending time-bound project completion [32], lack of interpersonal relationships [33] and lack of data/adequate information [34,35]. There are impacts on socio-environmental issues including living conditions and mass gatherings. There are impacts on persons with special needs including elderly issues, absence of social security measures and lack of access to essential services. COVID-19 affects religious practices, particularly from the closure of places of worship and cancellation of religious services [36].

### 4.2. Emerging Trends in Multi-Stakeholder Participation

The literature review highlights emerging trends in multi-stakeholder participation including global preparedness/contingency planning, the lack of post-crisis reconstruction and recovery, the presence of weak legal and institutional mechanisms, weak infrastructural facilities including communication networks, the lack of systematic and periodic assessment, the lack of accounting of potential losses, and the presence of poorly managed financial, technical and human resources. Other challenges include managing spontaneous behavioural reactions e.g., generalised panic, rumours/conspiracies regarding the spread of COVID-19, exposure to the elements (living conditions) and availability of good food and nutrition to fight against COVID-19. Furthermore, there is a need to work for vaccination, rehabilitation, water supply, food safety, basic sanitation, personal hygiene, research into other zoonotic diseases and investment in research and development [37]. 

### 4.3. Enhancing Nationwide Preparedness and Responses

One of the challenges involved in the process of nationwide preparedness to fight against COVID-19 is that it can lead to a national emergency [38]. This calls for dedicated funding for staffing, equipment and resources and resource allocations that are needed to support state, local and other health departments. Strengthening the linkages among all levels of state and non-state actors is very important to ensure multi-stakeholder participation in handling this pandemic (Table 1) [39].

From the above Table 1, we can understand the approaches, methods and strategies followed by different countries. For example, Taiwan used networking, proactive testing, border control and transparency as a method to handle the situation. They had frequent health check-ups, public education and relief measures to business as strategies to manage the crisis. Using multi-layer governance, they partnered with local government, private organisations, insurance companies and citizens to manage the COVID-19 situation [40,41,42,43,44]. South Korea used timely emergency response and a nationwide framework of networks of stakeholders as a method and, as a strategy, they followed shared interest, priority-based emergency responses and rapid response, partnering with government, community and chief scientific officers [45,46,47,48,49,50]. China and Singapore used the collaboration of scientists including social sciences as a method. As part of this strategy, they used large-scale coordination as an institutional and timely response and partnered with government, industry, banks and financial institutions and worked for community resilience [51,52,53,54,55]. 

The USA used networking as a basic method; and outbreak management and infection control as their strategy. They partnered with multi-layer government, private industries and companies and CSOs (Civil Society Organisation) [56,57,58,59]. Malaysia used movement control orders as a method and followed these steps with their strategies including producing more PPE (personal, protective and equipment), fundraising, collaboration with healthcare service providers and inducting additional laboratories. They partnered with government, CSOs and the community [60,61,62,63]. India used lockdown as a method and used emergency management and interstate transmission control as strategies to manage the situation. They partnered with multi-layer government, private sector organisations and CSOs [56,64,65,66,67]. Italy used pandemic management as a method and institutional arrangements as their strategy. They partnered with government and CSOs [68,69,70,71,72,73]. 

Turkey used lockdown and proactive policy for their method. They used rapid and strong response, extensive use of institutional resources and factual information campaigns as their crisis management. They partnered with government, community and the religious leaders [74,75,76]. Canada used social distancing, travel restrictions, integration of social sciences as their method. They used well-functioning federalism, long term care, rapid testing and tracing, face mask mandates as their crisis management; partnered with multilayer government [77,78,79,80].

France used nationwide lockdown as their method, using closure of non-essential public places and services, internal and international travel restrictions, cancellation of public events, care categorization and anticipation strategy as their crisis management, partnered with government and health department [55,81,82,83,84].

Japan used emergency (sub-national and local) as their method. They used effective implementation of self-discipline, avoiding “Three Cs” (closed spaces with insufficient ventilation, crowded conditions with people, and conversations at a short distance), no lockdown, recommendations regarding closure of schools and work places and public information campaigns as their crisis management. This was partnered with government and community [40,55,85,86,87,88].

Sweden used pandemic management—long term plan as their strategy. They used temporary bans on nonessential travel, recommendations on social distancing, working online, voluntary self-protection, non-closure of gyms, schools, restaurants and shops as their crisis management. This was partnered with government, voluntary organizations, and community [55,89,90,91,92,93].

Germany used social lockdown & economic lockdown as their method. They used national–wide social distancing and contact restrictions, personal care business centres were closed (hair dressers, tattoos, massage centres, etc.), different states followed different styles of lockdown for example strict lockdown—stay at home order and lenient lockdown—not to leave the house without a reason, closure of churches, recommendation on wearing of face masks, good medical preparedness, developed a reliable testing system, stock of testing kits, early testing and tracing as their crisis management. They partnered with government (National & Federal state), public and private hospitals, medical professionals, virologists, public health experts, laboratories, community, self-discipline, and citizens [94,95,96,97,98,99].

New Zealand used lockdown as their method. They used lockdown measures, closure of schools, non-essential workplaces, travel restrictions, restrictions on social gathering, social distancing, border control, rapid and science-based risk assessment, rapid testing and contact tracing, community transmission control measures, promotion of hand washing hygiene, medical preparedness, arranged more ICU & ventilator facilities and safeguarding healthcare professionals as part of their crisis management. They partnered with government, public and private hospitals, medical professionals, virologists, public health experts, laboratories, community, self-discipline and citizens [100,101,102,103,104].

It is evident that, different countries adopted various strategies to accommodate multi-stakeholder participation to handle the pandemic and its impact on lives. The approach to method and strategies varied upon the number of cases, available resources and the socio-political structure of the country.

## 5. Policy Announcement from Selected Countries for COVID-19

National Level COVID-19 Public Health responses included international travel restrictions, improving health facilities, setting strict following quarantine rules, guidance and compliance; tracking and testing, building up advisory systems, creating public awareness, controlling non-essential businesses, strengthening government services, restrictions on mass gathering, closure of schools and universities and imposing curfews. Some countries implemented good health data management/epidemiological databases, declared a state of emergency, imposed internal travel restrictions, implemented lockdown policies and followed decentralised communication as shown in Table 2. 

While others made the community be proactive, coordinated the works with clear role clarity, coordinated different policies, shared responsibilities and implemented effective public health measures. Some connected with their stakeholders by establishing mutual trust and through clinical manifestation to manage COVID-19 [40,45,47,51,56,60,68,105,106,107,108,109,110,111,112,113,114,115,116,117,120,121,122,123,124,125,126,127,128,129,130,131,132]. 

### Strategies Followed to Combat COVID-19

Various countries followed different strategies like extensive testing, contract tracing, community mobilisation, crisis precautions, cluster containment strategy, public health surveillance, proactive state leadership, proper planning, knowledge of COVID-19, expect the unexpected, creating awareness, service orientation and supply chain information to fight against COVID-19 (Table 3). 

Lessons learned from different countries involve the strengthening of crisis management and response strategies, increasing efforts to recognise cognitive bias and avoid partial solutions. Learning is critical and a readiness to accept the limitations is necessary. Understanding that extensive testing of symptomatic and asymptomatic cases early and proactive tracing of potential positives is very important. A strong emphasis on home diagnosis and care, specific efforts to monitor and protect health care and other essential workers, and collecting and disseminating data are important, as well as the resilience of affected/infected individuals [151]. It is important to address the plight of farmers, labourers and workers towards social protection measures. Health departments should concentrate on the robust collection of health data and epidemiological databases (for health policies and to ensure public health surveillance). The government should recognise the role of local international non-governmental organisations (INGOs) to the pandemic response and encourage timely provision of medical supplies and hygiene kit to individuals. The government should focus on the provision of social support and care to appropriate communities and vulnerable populations, co-ordination of funding activities and volunteers, R&D in life-saving medical innovations and to Test, Test and Test again the people in order to bring COVID-19 under control [40,45,48,51,56,60,68,69,123,124,125,126,127,128,129,130,133,134,135,136,137,138,139,140,141,142,143,144,145,146,147,148,149,150,151]. 

## 6. Discussion

The paper has presented different strategies, policies and methods used by different countries to fight against COVID-19. There is no one solution that can solve COVID-19, but through multi-stakeholder participation it is possible to find the most appropriate strategies to fight against COVID-19. Countries need to identify innovative and culturally acceptable measures to combat this crisis. Efforts should be taken to identify easily available, culturally adaptable local technology that is accessible and affordable to everyone. There is a need to address the immediate and long-term impacts of COVID-19 [152]. In pandemic times, there must be promotion of culturally acceptable strategies for physical distancing coupled with social solidarity [153]. There is a need to advocate for the advancement and strengthening of social welfare services as an essential protection against the pandemic [154]. There is a need to develop capabilities at all levels for emergency and pandemic prevention and management where each stakeholder’s strength and skills are identified, targeted and harmonised within general response and management systems [155].

There is a need to strengthen inter-organisational coordination, participation, accountability and local responsibility with central coordination to handle the pandemic impact effectively [156]. Societies also need significant resources and dedicated funding to deal with emerging and re-emerging infectious diseases focusing on its future recurring possibilities, prevention and management [157]. There should be incentives given to people for early reporting [158] followed by developing strategies to prevent antimicrobial resistance [159,160]. 

The health impact of recent outbreaks should be properly studied and there is a need to communicate effectively with public health emergency management including hazard and risk assessment, prevention and mitigation, incident management, resource management, communications, operations and training, exercising evaluation, corrective action and quality improvement [161]. Government should focus on the impact of sudden job losses and depletion of income due to COVID-19 and acute hardships for millions of urban and rural households, especially those working in the informal sector with no contracts, including migrants. Governments should find solutions to the complex challenges of health and nutrition, poverty, hunger and acute undernourishment of several million people, rising domestic conflict, violence and depression. Major economic problems like a reversal in capital follow as global risk, oil market deep-diving into negative, economic stagnation and the plight of labour, require further attention. Governments must also address the risk of health inequalities especially in vulnerable groups [162,163,164].

### Importance and Implications of Public Policies

While communicating to people there should be credible communication to the public without politicising the message [165]. Countries should come together, even if digitally/virtually, in order to take bold action since the virus knows no borders [166]. The public sector must lead society with a global approach to mitigate the impact of COVID-19. This involves public health emergency actions, identifying economic impacts, and combating misinformation and disinformation about the disease and its spread (Harvey, M. Whole of Society Approach [167]). Governments should focus on providing authoritative information via multiple sources to ensure accurate data, to slow the spread so that our health systems are not over-stressed (Kayyem, J. Disruption is the Plan [167]). There is a need to encourage increasing transparency, impose control measures and appropriate restrictions, design suitable prioritisation guidelines regarding the allocation of scarce resources and make use of effective technologies (Saghafian, S. Transparency, Control, Prioritization [167]). Countries should strive to recognise the potential for psychological burnout from long hours of work and potential demoralisation from persistent stress (Howitt, A.; Leonard, H. Energetic Mobilization [167]). Governments need to strike a balance between protecting the health of people and respecting human rights (Sikkink, K. Rights and Responsibilities [167]); to invest in vaccine and therapeutics against COVID-19 (Chandra, A. Vaccine Investment [167]); and to identify new priorities and revisit national spending priorities (Bilmes, L.J. How the Public Sector and Civil Society Can Respond to the Coronavirus Pandemic: New Priorities [167]). The government should address the long-standing challenges of health and nutrition of low-income households [168]. Governments must create synergy between partners and encourage collaboration to identify and engage in strong partnerships. 

## 7. Suggestions for Effective Interventions

Despite the breadth of this study, we are not presenting generalised suggestions for the most effective interventions, as there is so much variation across contexts, cultures and climates, and no single approach is most appropriate in all cases. Instead, we present the multi-stakeholder participation model as one of the appropriate models to be implemented in combating COVID-19. We need to create effective mechanisms through which to enable collaboration between international, national and regional organisations, and we should strive to establish pathways through which multiple actors can work together [169] and create synergy among society, economy and development [170]. An understanding of pandemic risks in all its dimensions, interlinking of disaster management and development planning is required [171]. There is also a need to encourage clinical and community-based research [172] and to strive to enhance healthcare data management for evidence-based research [173,174]. Successful interventions always assess the felt need of the community and then, through active and effective legal enforcement as required, facilitate and enable education to create a context of personal and public accountability and social responsibility. Self-discipline is one of the better interventions through which we can fight against COVID-19 so this can be achieved successfully [40]. The most effective intervention may be a combination of the different suggestions presented according to the needs, wants and situation of each country.

### Scope for Future Research

There is a need to better understand the COVID-19 crisis life cycle [175], and more research is required to know the causes and consequences (recovery, mitigation, response and preparation). Further analysis can be done by revisiting datasets, redefining relevant methodologies, facilitating access to online resources and exploring culturally relevant approaches. There is a need to improve access to relevant information sources and compile robust data of active and closed COVID-19 cases and their relatives. We need to evolve a global monitoring framework and find ways to implement the sustainable development goals [176]. Additional work is required to explore COVID-19’s impact on social development, human happiness and well-being of professionals, carers, their families and others in the community. Evidence must be synthesised more rapidly and it is needed the provision of large-scale intervention guidelines and longer-term strategies for human happiness, well-being, social and economic recovery. Further work is required to ensure adequate quality of research work and to better communicate the findings with multi-stakeholders, including policy briefs. There is a need to strengthen community-based crisis risk management, replicate best practices and learn from the field of diverse multispectral partnerships [177].

## 8. Limitations

Although the present study has accomplished some significant and interesting results, there are certain research limitations and challenges that can be improvised for better research in this field. First, due to the lack of available consistent data on global pandemic COVID-19 multi-stakeholder participation in diverse aspects, it took a lot of time to collect and finalise the data sets. Second, significant differences in various technical subjects (e.g., SDSS) led to challenges in identifying the real current situations. Third, due to the lockdown, work restrictions and lack of full physical access to the universities, some library facilities were not available for the data search. This is to be a major limitation and could be better addressed in future research. Finally, during the data collection, some organisations, particularly for government organisations, did not respond within the time frame. However, most of the vital information was obtained during the stipulated data collection period.

## 9. Conclusions

The current study assessed the global literature regarding COVID-19 and disasters scientifically using Kantamaneni’s [20] methodology. Accordingly, the present study critically synthesises the data from diverse countries to effectively understand the strategies and methods used to manage pandemics. Results and discussion identified that multi-stakeholder participation is one of the most effective solutions to combat the COVID-19 pandemic and its impact on livelihood in the current situations. Moreover, results also explored that the amalgamation of Multi-Stakeholder and Spatial Decision Support System (MS-SDSS) has proven to be the most applicable model to identify the potential pandemic sources and to control the spread of it across the world. While transdisciplinary approaches to problem structuring and decision-making to combat COVID-19 seem extremely promising, the conceptual MS-SDSS can bring out a synergic relationship between multi-stakeholders and help inform decision making in crisis management. This paper also promotes the need to strengthen public health surveillance and preparedness for pandemic management, through research, capacity building and action. The review study suggests that governments partner with collaborating institutions and provide support in surveillance, preparedness and capacity building during public health emergencies. The study also encourages an inclusive, innovative community-based approach, (including virtual and home-based care). Cumulatively, the current study results help the researchers, diverse stakeholders, policy and decision-makers to continue further research or work on this topic.

## Figures and Tables

**Table 1:** Case synthesis of multi-stakeholder activities to control COVID-19.

Country/Territory	Method/Approach	Crisis Management	Partners/Stakeholders	Source
Taiwan	Networking, proactive testing, border control, transparency	Frequent health check-ups, public education, relief to business, use of information technology	Multi-layer governance, private organisations, insurance companies, citizens	[40,41,42,43,44]
South Korea	Timely emergency response, the nationwide framework of networks among stakeholders	Shared interest, priority-based emergency response, rapid response, effective anti-COVID-19 measures, rapid testing, effective isolation strategy, scaling up resources, use of information technology	Govt, community, CSOs (Civil Society Organisation)	[45,46,47,48,49,50]
China, Singapore	The collaboration of Science including Social Sciences	Large scale coordination, institutional timely response, community resilience, national level response, effective contact tracing	Govt, industry, banks and financial institutions	[51,52,53,54,55]
USA	Networking	Outbreak management, control, ineffective response to the COVID-19	Multi-layer Govts, private, CSOs	[56,57,58,59]
Malaysia	Movement control order	Produce PPE (personal, protective equipment), fundraising, collaboration with healthcare service providers, inducting additional Labs, effective testing and contact tracing, effective communication and daily briefings	Govt, CSOs, community	[60,61,62,63]
India	Lockdown	Emergency management, interstate transmission control, laboratory network, ineffective practice of physical distancing, closure of educational institutes	Multi-layer Govts, private, CSOs	[56,64,65,66,67]
Italy	Pandemic management, lockdown	Institutional arrangements, undermining the virus, triple “Ts” (testing, tracing and treatment)	Govt, CSOs	[68,69,70,71,72,73]
Turkey	Lockdown, proactive policy style	Rapid and strong response, extensive use of institutional resources, factual information campaigns	Presidential system of government, community, religious authorities	[74,75,76]
Canada	Social distancing, travel restrictions, integration of social sciences	Well-functioning federalism, long term care, rapid testing and tracing, face mask mandates	Multilayer government	[77,78,79,80]
France	Nationwide lockdown	Closure of non-essential public places and services, internal and international travel restrictions, cancellation of public events, all covid-19 system—care categorization and anticipation strategy	Govt, health department	[55,81,82,83,84]
Japan	Emergency (sub-national and local)	Effective implementation of self-discipline, avoiding “Three Cs” (closed spaces with insufficient ventilation, crowded conditions with people, and conversations at a short distance), no lockdown, recommendations regarding closure of schools and work places, public information campaigns	Govt, community	[40,55,85,86,87,88]
Sweden	Pandemic management—long term plan	Temporary ban on nonessential travel, recommendations on social distancing and working online, voluntary self-protection, non-closure of gyms, schools, restaurants and shops	Govt, voluntary organizations, community	[55,89,90,91,92,93]
Germany	Social lockdown & Economic lockdown	Nation–wide social distancing and contact restrictions, personal care business centres were closed (hair dresses, tattoos, massage centres, etc.), different states followed different styles of lockdown e.g., strict lockdown—stay at home order and/or lenient lockdown—not to leave the house without a reason, closure of churches, recommendation on wearing of face masks, good medical preparedness, developed a reliable testing system, stock of testing kits, early testing and tracing	Govt (National & Federal state), public and private hospitals, medical professionals, virologists, public health experts, laboratories, community, self-discipline, citizens	[94,95,96,97,98,99]
New Zealand	Lockdown	Lockdown measures, closure of schools, non-essential workplaces, travel restrictions, restrictions on social gathering, social distancing, border control, rapid and science-based risk assessment, rapid testing and contact tracing, community transmission control measures, promotion of hand washing hygiene, medical preparedness, arranged more ICU & ventilator facilities, safeguarding healthcare professionals	Govt, public and private hospitals, medical professionals, virologists, public health experts, laboratories, community, self-discipline, citizens	[100,101,102,103,104]

**Table 2:** Policy announcements for COVID-19.

International travel restrictions [105,106]
Improving health facilities [107]
Strict quarantine measures [108,109,110]
Tracking and testing [47,108,111,112,113]
Built new hospitals for the treatment COVID-19 [110]
Building up advisory systems and Creating public awareness [47,114]
Stoppage of Non-essential businesses [108,112]
Strengthening Government services [115,116]
Restriction on mass gathering [108,112,116]
School and university closure [108,109,112,116]
Curfew [109,112,117]
Health data management/ epidemiological data base [47,108,114]
State of emergency [108]
Internal travel restriction [112]
Lockdown policy [111,112,117,118,119]
Decentralised communication [47]
community to be proactive, sharing of responsibility [120,121]
Stakeholders and clinical manifestation of COVID-19 [45,122]

**Table 3:** Case synthesis of lessons learned from the experience of different countries.

Lessons Learned from the Experience of Different Countries
1. Strengthen crisis management and response strategies [133]
2. Recognize your cognitive biases [134]
3. Avoid partial solutions [69]
4. Learning is critical [48]
5. Extensive testing of symptomatic and asymptomatic cases early on [135]
6. Proactive tracking of potential positives [136]
7. A strong emphasis on home diagnosis and care [137]
8. Specific efforts to monitor and protect health care and other essential workers [138]
9. Collecting and disseminating data is important [139]
10. The resilience of affected/infected individuals [140]
11. Awareness of the plight of farmers, labours [141]
12. Social protection measures [142]
13. A robust collection of health data, and epidemiological database (for health policies) [143]
14. To ensure public health surveillance [144]
15. Recognition of the role of international non-governmental organisations (INGOs) [145]
16. Timely provision of medical supplies and hygiene kit [146]
17. Provision of social support and care to the appropriate communities and vulnerable populations [147]
18. Coordination of funding activities and volunteers [148]
19. R&D in life-saving medical innovations [149]
20. Test, Test and Test again [150]
